# A Comparative Evaluation of Intra-articular Bupivacaine vs Bupivacaine and Dexmedetomidine for Postoperative Analgesia in Arthroscopic Knee Surgeries

**DOI:** 10.7759/cureus.43956

**Published:** 2023-08-23

**Authors:** Amreesh Paul, Anjali Borkar, Nikhil Bhalerao, Dnyanshree Wanjari

**Affiliations:** 1 Anaesthesiology, Jawaharlal Nehru Medical College, Datta Meghe Institute of Higher Education and Research, Wardha, IND

**Keywords:** intra-articular injection, postoperative analgesia, orthopaedic arthroscopic surgery, 0.5% bupivacaine, dexmedetomidine

## Abstract

Background

The study was done to determine the duration of postoperative analgesia brought on by incorporating intra-articular Bupivacaine with Dexmedetomidine as an adjuvant following knee arthroscopies.

Methods

A prospective randomized control study was conducted on 60 patients of ASA classes I and II, between the ages of 20 and 60 years, undergoing arthroscopic surgeries of the knee under spinal anaesthesia. The patients were divided into group B and group D, each containing 30 patients. The participants in group B were administered Inj. Bupivacaine 0.5% 19 mL + 1 mL of normal saline intra-articularly and the participants in group D were administered Inj. Bupivacaine 0.5% 19 mL, Inj. Dexmedetomidine 1 μg/kg and normal saline post-surgery. The number of analgesics used in the first 24 hours, pain levels using the visual analogue scale and the timing of administration of the first analgesic dose between the two study groups were evaluated.

Results

In comparison to the Bupivacaine group, the Dexmedetomidine group required fewer rescue analgesics. The visual analogue visual scale score in group B at four hours and six hours was 2.7 ± 1.39 and 2.9 ± 1.03, respectively, and in group D at four hours and six hours was 1.9 ± 1.09 and 1.83 ± 0.91. The visual analogue scale scores at these times were statistically significant. The visual analogue scale scores at 12 hours and 24 hours were statistically not significant.

Conclusion

Dexmedetomidine added to Intra-articular Bupivacaine provides an increased duration of postoperative analgesia in patients undergoing arthroscopic surgeries of the knee. The combination offers improved analgesia and reduces the overall dosage of rescue analgesics needed without causing substantial side effects.

## Introduction

Pain following surgery, if inappropriately treated, leads to delayed recovery. The Joint Commission on Accreditation of Healthcare Organizations identifies pain as the fifth vital sign in addition to pulse rate, blood pressure, temperature, and respiratory rate [1]. Therefore, pain control post-surgery is just as crucial as managing pain during surgery [2]. An anaesthesiologist must know that postoperative pain results from several neurophysiological interactions rather than actual tissue damage. As a result, postoperative analgesia is significantly more challenging, and the perfect pain management strategy is still elusive. Analgesics provide comfort while reducing sympathetic nervous system reaction, preventing tachycardia and hypertension [3]. When pain receptors are triggered, they produce chemicals known as neurotransmitters. The neurotransmitter is glutamate, also called the substance P [4]. The term “nociception” refers to the entire pain-transmission mechanism. Nociceptive pain receptors produce afferent nerve conduction in the peripheral nerve fibres. These communicate stimuli to the brain and spinal cord [5]. A-delta fibres and C-sensory fibres are the two main types of myelinated neurons identified in afferent nociceptors in tissues. These swiftly transmit pain impulses to the spinal cord, which is processed in the ventral posterior thalamus nucleus before transmitting to the cerebral cortex in the brain. Additionally, they use the lateral spinothalamic tract to transmit the body's response to pain to the brain stem, specifically the thalamus [6].

Arthroscopy offers a minimally invasive alternative to conventional open surgical procedures, which frequently require extensive incisions to enable sufficient joint exposure [7]. Reduced comorbidity, speedier rehabilitation, and socioeconomic improvements are among the benefits that are supported by evidence [8]. Even though arthroscopic knee procedures result in a quicker recovery, the pain they cause can be excruciating right after surgery. Hence, many pain relief modalities are used [9]. Local anaesthetic administration intra-articularly with or without adjuvants is one such technique that provides excellent analgesia in the initial postoperative period [10]. In recent years, arthroscopic knee procedures have become highly popular worldwide. Intra-articular analgesics can be utilized to lessen pain severity. Effective, safe, and feasible postoperative analgesia is essential. Following surgery, non-steroidal anti-inflammatory drugs (NSAIDs) and opioids are given to reduce discomfort. Nevertheless, they do have some limits [11].

Bupivacaine developed in 1957, is primarily utilized in local infiltration, neuraxial blockade and regional anaesthesia. Local anaesthetics inhibit the generation of action potentials as they increase the nerve cell excitability threshold [12]. Local anaesthetics' effectiveness, duration of action, and plasma protein binding are all influenced by lipid solubility. Local anaesthetics access nerve fibres on a neutral-free basis. Conduction is prevented by the interaction of cationic and ionised forms on the sodium channel's inner surface. Additionally, local anaesthetics with lower Pka initiate their actions more quickly. Action potentials can move through axons, dendrites, and muscle tissue owing to sodium channels that are membrane proteins. One large alpha subunit and one or two small beta subunits make up sodium channels in various tissues [13]. All three local anaesthetic binding and ion conduction sites have four domains with six alpha-helical membrane-spanning segments. The glycosylation of the exterior surface of the alpha subunit enables the orientation of the channel inside the cytoplasmic membrane. Tetrodotoxin and scorpion venom bind to the extracellular sodium channel, in contrast to local anaesthetics [14]. Furthermore, local anaesthetics induce dose-dependent myocardial depression [15].

Dexmedetomidine has sedative, analgesic, and anaesthetics-sparing properties and causes sympatholysis when administered systemically [16]. Evidence also points to a reduction in pain from arthroscopic procedures when Dexmedetomidine is administered intra-articularly, alone or in conjunction with other medications. Dexmedetomidine targets peripheral nociceptive receptors and can be used as an adjuvant to Bupivacaine after the surgery to provide sufficient analgesia [17].

The aim of the study was to analyze the duration of post-operative analgesia brought on by intra-articular Bupivacaine vs Bupivacaine and Dexmedetomidine after arthroscopic knee surgeries. The primary objective was to evaluate the duration of postoperative analgesia by using Dexmedetomidine as an adjuvant to intra-articular Bupivacaine ensuing knee arthroscopies. The secondary objectives were to evaluate the analgesic quality, the total doses of rescue analgesics required during the first 24 hours after the intra-articular administration, and the incidence of side effects when Dexmedetomidine is injected intra-articularly with Bupivacaine. The generated hypothesis of this study was that when Dexmedetomidine is used as an adjuvant to Intra-articular Bupivacaine at the end of knee arthroscopies, it may be useful in the prolongation of the duration of postoperative analgesia without causing a negative impact on patient safety.

## Materials and methods

This prospective randomized control study was done at the Department of Anesthesiology, Jawaharlal Nehru Medical College (JNMC), Datta Meghe Institute of Higher Education & Research (DMIHER), Sawangi, Wardha, between January 2021 and January 2022, after the approval from the Ethics and Screening Committee of JNMC, DMIHER, Sawangi (M), Wardha. Patients undergoing arthroscopic knee surgeries meeting the inclusion and exclusion criteria were enrolled for the study. The patients were evaluated one day before the scheduled date of surgery. A thorough history was obtained from the patient, and systemic and general examinations were done. Cardiovascular, neurological or respiratory problems were ruled out. The patient's height, weight, and body mass index were noted down, and routine investigations were done and noted. The study methodology was discussed with the patient in detail, along with the risks, benefits, and associated complications. The Visual Analogue Scale (VAS) was then explained to the patient, with zero on the scale as no pain and 10 on the scale as excruciating pain. Informed written consent was procured from all participants.

Inclusion and exclusion criteria

Patients between the age of 20 and 60 years, with American Society of Anesthesiologists (ASA) class I and II, posted for arthroscopic surgeries of the knee of duration between 90 and 130 min, were the participants in the study. Patients with obesity, elevated renal and hepatic parameters, heart disease, systemic hypertension, narcotic or NSAID intake before surgery, carcinoma, sepsis, coagulation disorders, and drug allergies were excluded.

The patients were kept nil per os from six hours for solids and two hours for clear liquids before the scheduled time of the procedure. Before the scheduled time of the surgery, they were shifted to the pre-operative room, and their vital parameters were noted down. The patients were allocated into the following groups using computer-based randomization, receiving 20 mL of the study drug intra-articularly. Group B received 19 mL of Inj. Bupivacaine 0.5% and 1 mL of normal saline after the completion of the surgery, and Group D received 19 mL of Inj. Bupivacaine 0.5%, Inj. Dexmedetomidine (1 μg/kg) and normal saline constitute a total of 20 mL after the completion of surgery. The study is triple-blinded, with the patient, the surgeon and the monitoring anaesthesiologist being unaware of the drugs that are administered.

**Figure 1:**
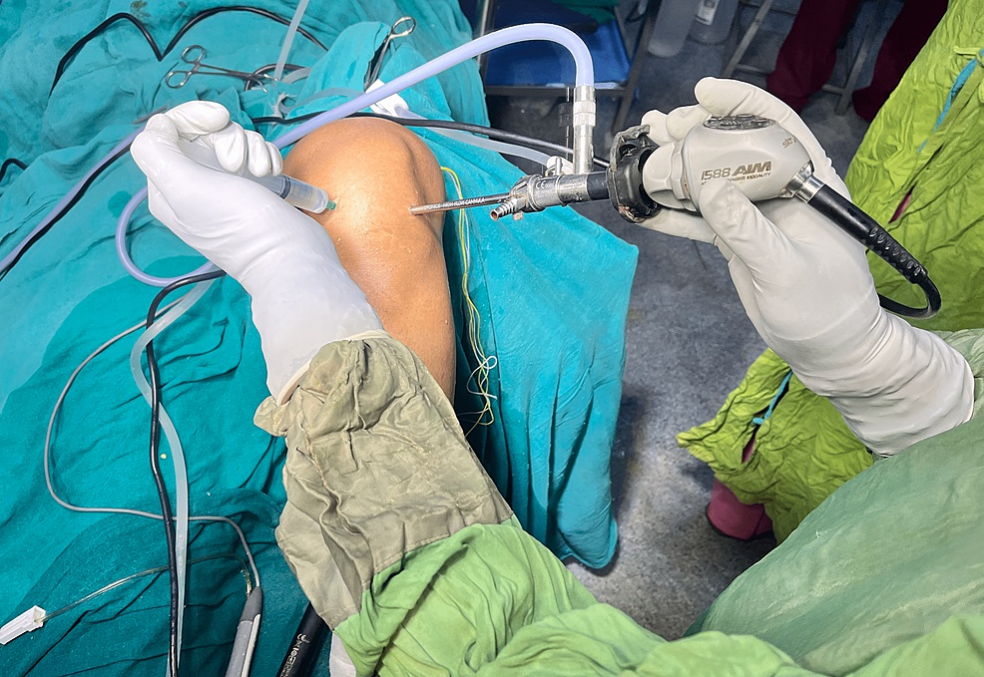
Intra-articular administration of study drug at the end of arthroscopic knee surgery 10 mins before the release of the tourniquet.

An 18-gauge intravenous cannula was inserted in the operating room, and ringer lactate infusion was started. The patient's baseline vital parameters were recorded using standard ASA monitors, and their baseline vital parameters were assessed. The methods of anaesthesia and the study were explained to the patient again. Under strict aseptic preparations, with the patient sitting, a dural puncture was done at L3-L4 interspace using a midline approach and a 25G Quincke's needle. After confirmation of needle placement in the intrathecal space by visualization of a good flow of CSF, 3 mL of 0.5% Inj. Bupivacaine (Hyperbaric) was instilled. Post spinal anaesthesia, the patient was made to lie down supine. The desired level of sensory block for the commencement of surgery was fixed to be T8 to T10 and was elicited by the pinprick method. A pneumatic tourniquet was attached to the thigh of the operative limb and was maintained with an inflation pressure of 250-350 mm Hg continuously during the procedure. Two-segment regression time was monitored for all patients and was noted down. After the surgery, the drugs for intra-articular administration were prepared in sterile conditions according to the group they belonged to and were handed over to the surgeon aseptically. The drugs were injected through the arthroscopy ports (Figure 1). The injection time was considered the study's starting point, and the tourniquet was deflated 10 min later.

An anaesthesiologist is not a part of the intra-operative team that monitored the patient in the post-operative period to avoid bias. Pain severity was assessed using the VAS score. Vital parameters were recorded at zero, one, two, four, six, eight, 12, and 24h. Patients requiring intraoperative rescue analgesics were excluded from the study. In the postoperative period, a VAS of ≥ 4 was considered significant and Inj. Paracetamol 1 g i.v. was given. The time of administration was noted down. VAS was evaluated at zero, one, two, four, six, 12 and 24h postoperatively, ranging from 0 to 10. When the VAS was more than 4, Inj. Paracetamol 1 g was administered intravenously and was repeated as required. Administration time of Inj. Paracetamol and the number of doses given were noted down. Side effects associated with the administration of Dexmedetomidine were noted down.

The postoperative monitoring included the time between the administration of the drugs intra-articularly till the administration of Inj. Paracetamol was considered as the duration of postoperative analgesia, visual analogue scale to assess the quality of analgesia during the first day post-operatively, the total doses of Inj. Paracetamol is required during the first day post-operatively, vital parameters including pulse rate, blood pressure, respiratory rate and oxygen saturation and complications like hypotension, bradycardia, nausea and vomiting, sedation, etc.

**Figure 2:**
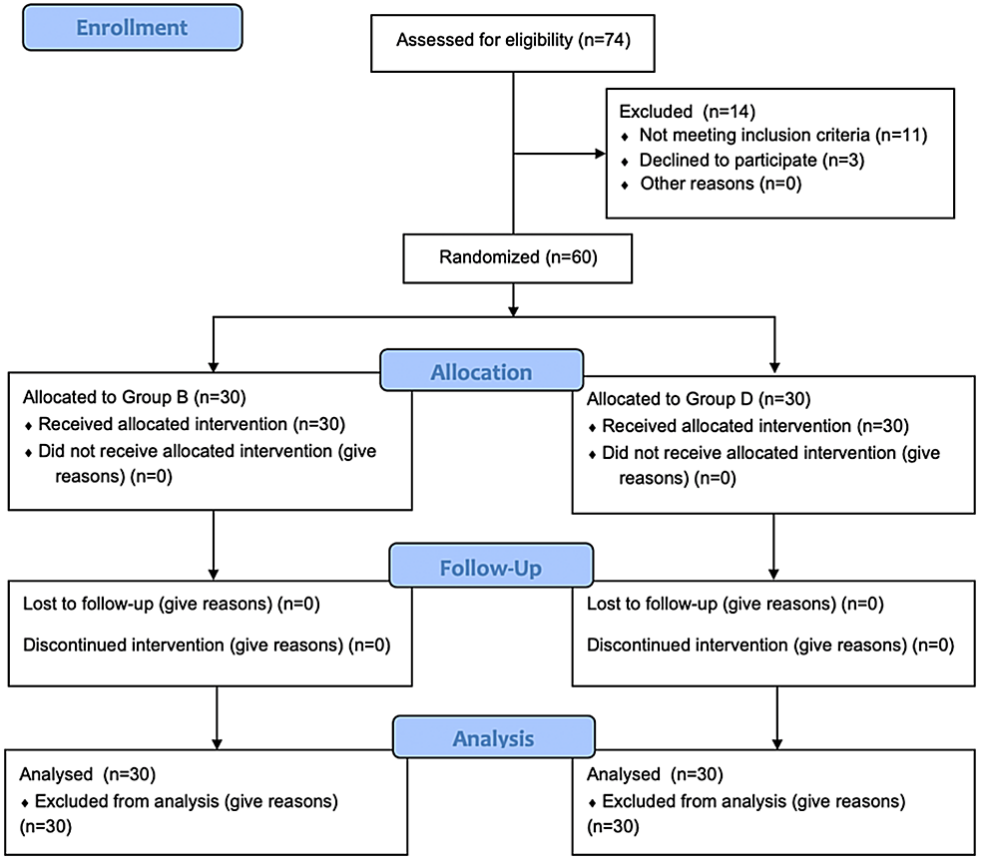
CONSORT flow chart of the study

Seventy-four patients were initially assessed to fulfil the inclusion criteria. Fourteen patients not meeting the criteria and those unwilling to participate were not enrolled. The study was conducted on the other 60 patients. The CONSORT flow chart of the study is shown in Figure 2.

Sample size calculation

The sample size was determined by taking the total consumption of rescue analgesia in milligrams in 24 hours with reference to the study by Mostafa et al. [18]. The reference study was done with 42 patients. The mean of rescue analgesics used in group B was 61.70 mg, and in group, BD was 21.40 mg with a standard deviation of 52.90 mg and 38.90 mg, respectively. The power of the study was established at 80%, the α error was set at 0.05, and the confidence interval was 95%. A sample size of 21 patients in both groups was needed to detect at least a 25% variation in the number of required analgesics in both groups. Since patients may drop out of the study midway, the sample size was increased to 30 patients per group.

Statistical methods

Mean ± standard deviation was used to express all the quantitative data, and percentages were used to represent all qualitative data. Chi-square was used to find the association between two categorical variables. Quantitative parameters, custom distribution, and the mean values were compared using an independent sample t-test or paired t-test. A p-value below 0.05 was regarded to be statistically significant.

## Results

A total of 60 patients from various walks of life were enrolled in our study. All of them were scheduled to undergo arthroscopic surgeries of the knee under spinal anaesthesia. Demographic data were collected from all patients and were analyzed. The comparison between both groups in terms of age, gender, height and weight was statistically insignificant, with a p-value of more than 0.05. As seen in Tables 1-4, the patients in both groups were comparable in terms of demographic data.

**Table 1:** Demographic data - age

Age (Years)	Group B	Group D	P-value
20-30	19	17	0.199
31-40	8	7	
41-50	3	5	
51-60	0	1	
Total	30	30	
Mean ± SD	28.77 ± 7.98	31.8 ± 9.99	

**Table 2:** Demographic data - gender

Gender	Group		Chi-square	P-value
	B (N=30)	D (N=30)		
Female	7 (23.33%)	3 (10%)	1.920	0.166
Male	23 (76.67%)	27 (90%)		

**Table 3:** Demographic data - height

Height in cms	Group B	Group D	P-value
140-149	1	0	0.083
150-159	6	3	
160-169	15	15	
170-179	5	11	
180-189	3	1	
Total	30	30	
Mean ± SD	164.83 ± 9.05	168.6 ± 7.44	

**Table 4:** Demographic data - weight

Weight in kgs	Group B	Group D	P-value
50-59	10	4	0.152
60-69	11	15	
70-79	8	8	
80-89	0	3	
90-99	1	0	
Total	30	30	
Mean ± SD	64.17 ± 9.56	67.47 ± 7.97	

Patients for whom the duration of surgery was less than two hours were enrolled for the study. If the duration of the surgery exceeded this time, the patients were excluded from the study. Table 5 shows the values of patients distributed according to the surgery duration in both groups. The mean duration of surgery in Group B was 109.43 ± 9.97 min, and in Group B was 111.03 ± 10.06 min. Most patients’ surgeries were between 100 and 120 min. Patients in both groups were comparable based on the mean duration of surgery (p = 0.538).

**Table 5:** Duration of surgery between groups

Duration of surgery in minutes	Group B	Group D	P-value
90-100	8	6	0.538
100-110	8	8	
110-120	8	8	
120-130	6	8	
Mean ± SD	109.43 ± 9.97	111.03 ± 10.06	

Table 6 compares mean arterial pressure between the groups at different time intervals. The difference between the two groups was non-significant based on mean arterial pressure at different time intervals till 24 hours postoperatively (p > 0.05). Patients were comparable in both groups concerning mean arterial pressure.

**Table 6:** Mean arterial pressure at different time intervals

Postoperative (hours)	Group B	Group D	P-value
0	94.19 ± 7.43	94.66 ± 6.65	0.799
1	95.14 ± 5.29	94.7 ± 6.38	0.770
2	95.02 ± 5.54	94.09 ± 6.79	0.562
4	96 ± 6.18	94.2 ± 6.71	0.284
6	93.93 ± 4.83	92.64 ± 6.11	0.369
12	93.46 ± 8.09	93.94 ± 4.46	0.779
24	98.8 ± 6.13	96.26 ± 3.39	0.051

Table 7 shows the comparison of pulse rates between the study groups at various intervals. The difference between the two groups was non-significant based on the mean pulse rate at different time intervals till 24 hours postoperatively (p > 0.05). Patients were comparable in both groups concerning mean pulse rate.

**Table 7:** Pulse rate at different time intervals between the study groups

Postoperative (hours)	Group B	Group D	P-value
0	79.9 ± 6.59	81.43 ± 6.83	0.380
1	79.97 ± 6.99	82.07 ± 7.75	0.275
2	79.9 ± 6.87	80.53 ± 7.74	0.739
4	85.13 ± 6.49	82.97 ± 8.01	0.254
6	84.93 ± 6.09	81.7 ± 7.8	0.079
12	81.57 ± 6.12	82.67 ± 8.57	0.569
24	80.67 ± 4.56	79.63 ± 6.02	0.457

Table 8 compares oxygen saturation between the study groups at different time intervals. The difference between the two groups was non-significant based on oxygen saturation at various time intervals till 24 hours postoperatively (p > 0.05). Patients were comparable in both groups concerning mean oxygen saturation.

**Table 8:** Oxygen saturation at various intervals

Postoperative (hours)	Group B	Group D	P-value
0	99.83 ± 0.46	99.77 ± 0.57	0.620
1	99.77 ± 0.57	99.77 ± 0.57	1.000
2	99.77 ± 0.5	99.63 ± 0.76	0.429
4	99.77 ± 0.5	99.7 ± 0.75	0.688
6	99.73 ± 0.58	99.7 ± 0.6	0.827
12	99.77 ± 0.5	99.73 ± 0.58	0.814
24	99.73 ± 0.58	99.67 ± 0.61	0.666

**Figure 3:**
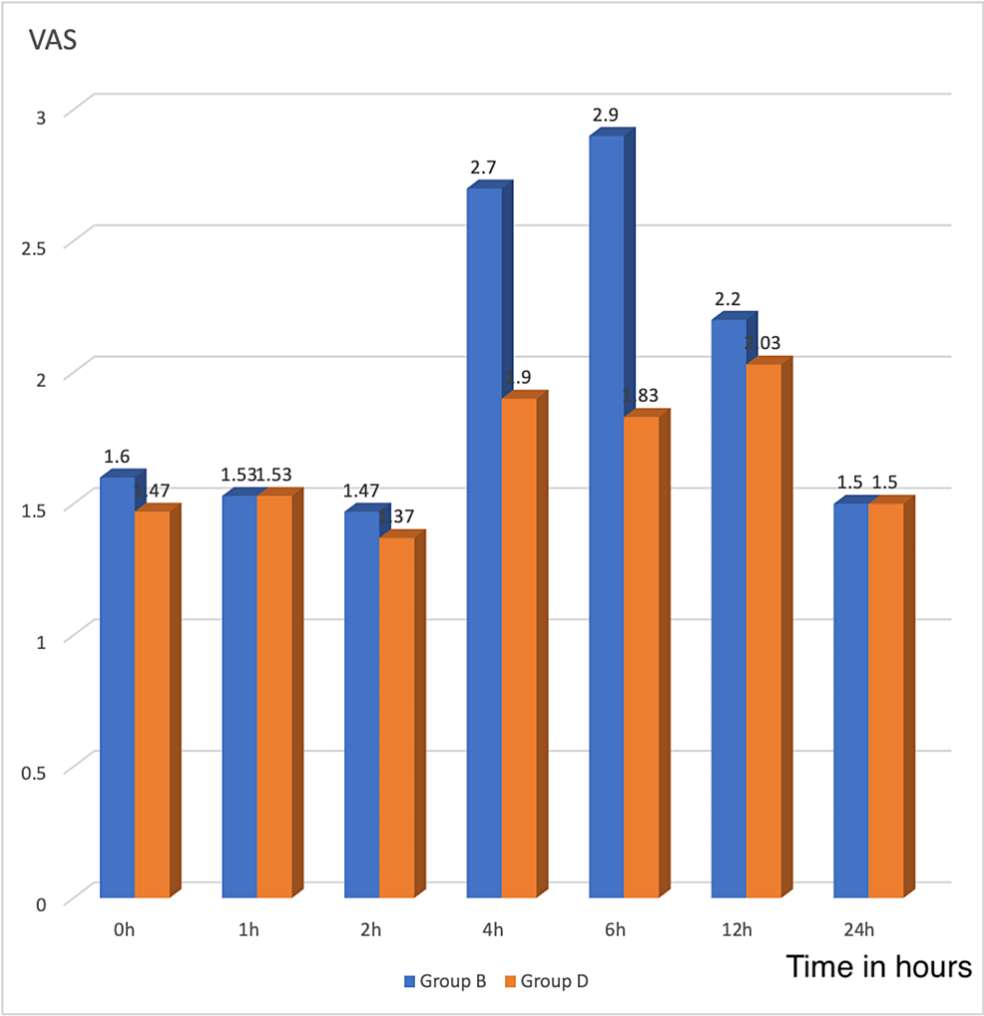
Bar chart showing the quality of analgesia at different time intervals. VAS - Visual Analogue Scale

Table 9 and Figure 3 compare the mean VAS score at various times between the study groups. Patients with a VAS score of more than four at any time interval were given Inj. Paracetamol 1 g and the time of administration were noted down. The VAS scores of the two groups during zero, one, two, 12, and 24h were statistically insignificant. Hence, the patients in both groups were comparable based on zero, one, two, 12, and 24h mean VAS scores (p > 0.5). In group B, the mean VAS during the fourth and sixth hours were 2.7 ± 1.39 and 2.9 ± 1.03, respectively, whereas, in group D, the mean VAS during the fourth and sixth hours were 1.9 ± 1.09 and 1.83 ± 0.91, respectively. This is statistically significant (p < 0.01).

**Table 9:** Comparison of quality of analgesia at different time intervals VAS - Visual Analogue Scale

Postoperative (hours)	Mean VAS Group B	Mean VAS Group D	P-value
0	1.6 ± 0.72	1.47 ± 0.63	0.449
1	1.53 ± 0.32	1.53 ± 0.46	1
2	1.47 ± 0.73	1.37 ± 0.56	0.553
4	2.7 ± 1.39	1.9 ± 1.09	0.016
6	2.9 ± 1.03	1.83 ± 0.91	<0.001
12	2.2 ± 1	2.03 ± 1	0.520
24	1.5 ± 0.68	1.5 ± 0.63	1.000

**Figure 4:**
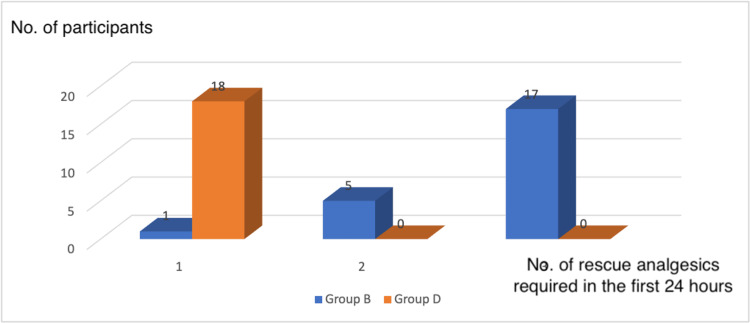
Bar chart showing the number of doses of rescue analgesics required in the first 24 hours post-operatively

Table 10 and Figure 4 show the distribution based on doses of analgesics administered on the first day post-operatively when VAS ≥4. In group D, 18 patients required rescue analgesics once in 24 hours as compared to only one in group B (p < 0.01). In the Dexmedetomidine group, no patient required two or three doses of analgesic compared to five and 17 patients requiring two or three doses of analgesics in the Bupivacaine group. This is statistically significant (p < 0.01).

**Table 10:** Number of doses of rescue analgesics required in the first 24 hours post-operatively

Number of doses of rescue analgesia required during the first 24 hours	No. of participants requiring rescue analgesics in Group B	No. of participants requiring rescue analgesics in Group D	P-value
1	1	18	<0.001
2	5	0	
3	17	0	
Mean ± SD	2.70 ± 0.559	1.00 ± 0.01	

Table 11 shows the time at which the first dose of rescue analgesic was administered, corresponding to the period of postoperative analgesia. The patients were administered Inj. Paracetamol 1 g as rescue analgesics, when the VAS score was more than four. In group D, 12 patients did not receive analgesics in the first 24 hours of the postoperative period. The mean time of administration was 273.13 ± 47.71 min in Group B and 477 ± 50.26 min in Group D. This is statistically significant (p < 0.001).

**Table 11:** Comparison of duration of postoperative analgesia

Parameter	Group (Mean ± SD)		P-value
	B (N=23)	D (N=18)	
Time of administration of analgesic (in minutes)	273.13 ± 47.71	477 ± 50.26	<0.001

## Discussion

The role of the modern anaesthesiologist has evolved from intraoperative care to comprehensive care. Postoperative pain management is equally as crucial as intraoperative pain management. Alpha 2 adrenoceptor agonist - Dexmedetomidine reduces pain by acting on peripheral, spinal, and supraspinal channels. Although it is believed that IA Dexmedetomidine exerts its analgesic effect primarily by direct local action, a central impact brought on by absorption into the systemic circulation cannot be explicitly ruled out. Similar to IA clonidine, IA Dexmedetomidine has the potential to act locally to block the transmission of nerve signals through C and A δ fibres, to provide analgesic effects by modulating the opioid analgesic pathway [19].

Local anaesthetics have severe adverse effects involving the central nervous system and the cardiovascular system. These typically result from unintentional intrathecal or intravascular administrations or an apparent overdose. Tongue numbness, lightheadedness, visual abnormalities, and muscle twitching are examples of central nervous symptoms of local anaesthetic toxicity that appear before cardiovascular symptoms and signs. More severe symptoms include seizures, coma, respiratory arrest, and cardiovascular depression. High doses cause sinus bradycardia and arrest by suppressing the sinus node's natural pacemaker activity. In our study, none of the participants experienced any adverse side effects. Thus, we assume that a 20 mL dose of Bupivacaine 0.5% administered intraarticularly is acceptable.

After arthroscopy of the knee joint, a single intra-articular injection of local anaesthetic has been advocated to provide adequate pain management and reduce the need for and potential side effects of systemic analgesics. Because of its prolonged duration of action, intra-articular Bupivacaine is frequently used. In our study, we evaluated the total number of analgesics used in the first 24 hours and the time of administration of the first dose of rescue analgesic between the two study groups. In the Bupivacaine group, one patient required one dose, five required two doses, and 17 required three doses of rescue analgesia in the first 24 hours after intra-articular administration of the study drugs. In contrast, in the Bupivacaine-Dexmedetomidine group, 18 patients required a single dose of rescue analgesic in the first 24 hours after intra-articular administration of the study drugs. Repeat rescue analgesics were not needed in this group. The p-value was <0.001, indicating statistical significance. The time of the administration of the rescue analgesics was 273.13 ± 47.71 min in the Bupivacaine group and 477 ± 50.26 min in the Bupivacaine-Dexmedetomidine group. The p-value was <0.001, indicating statistical significance. This is in accordance with the studies conducted by Mostafa et al., Avci et al., and Mooen et al. [18,20,21].

Vital parameters were measured at zero, one, two, four, six, 12, and 24h after the administration of the study drugs intra-articularly. All vital parameters were compared and found insignificant at all times in the study. These findings are consistent with the studies conducted by Mostafa et al., Salem et al., Mooen et al., and Avci et al. [18,20-22].

Salem et al. compared Dexmedetomidine and Fentanyl as an adjuvant to Bupivacaine in knee arthroscopies for post-operative analgesia. The authors concluded baseline VAS-s and VAS-d scores were comparable across the three groups. Regarding the postoperative VAS-s and VAS-d scores: At 30 min, one, two, four, and six hours, groups BF and BD showed significantly lower postoperative VAS-s and VAS-d scores than group B (P< 0.0001). However, group BF and BD were comparable at the same time points (p > 0.05). At four hours, eight hours, and 12 hours, group B's VAS-s and VAS-d scores were higher than those of the other two groups (p < 0.0001). There were no statistical differences between the three groups at 18 or 24 hours [22]. In our study, the patient's VAS score was noted at zero, one, two, four, six, 12, and 24 h post-intra-articular injection of study drugs. In our study, the residual effect of spinal anaesthesia was a factor for no statistical significance between the groups in the immediate post-surgery period. Once the impact of the spinal anaesthesia started wearing off, there were significant changes in the VAS scores in both groups. The VAS score in group B at four hours and six hours was 2.7 ± 1.39 and 2.9 ± 1.03, respectively, and in group D at four hours and six hours was 1.9 ± 1.09 and 1.83 ± 0.91. The VAS scores at these times were statistically significant. The VAS scores at 12 hours and 24 hours were statistically not significant.

Mostafa et al. concluded that no side effects were observed in the Bupivacaine group. In the Bupivacaine-Dexmedetomidine group, two patients (5.7%) had hypotension, and three (8.5%) had bradycardia [18]. Similarly, in our study, no side effects were observed in the Bupivacaine group. In contrast, in the Bupivacaine-Dexmedetomidine group, two patients (6.67%) had bradycardia, and two (6.67%) had hypotension. The side effects associated with the administration of drugs intra-articularly had no statistical significance with a p-value of 0.112.

The mean age of our study participants in the Bupivacaine group was 28.77 ± 7.98 years, and the Dexmedetomidine group was 31.8 ± 9.99 years. The patients' mean age was statistically insignificant, with a p-value of >0.05. In comparison, Mostafa et al. reported the mean age of patients in their study to be 35.17 ± 9.78 years and 34.89 ± 9.22 years in Bupivacaine and Bupivacaine Dexmedetomidine group [18]. This is in accordance with our research. Table 2 (graph 2) shows the distribution of patients according to gender. It was observed that male patients outnumbered female patients in both groups. In our study, there were 23 male and seven female patients in group B and 27 male and three female patients in group D were included in the study. The patients were comparable in both groups with respect to gender with a p-value of 0.116.

The mean height of our study participants in the Bupivacaine group was 164.83 ± 9.05 cm, and the Dexmedetomidine group was 168.6 ± 7.44 cm. The mean weight of our study participants in the Bupivacaine group is 64.17 ± 9.56 kg, and in the Dexmedetomidine group is 67.47 ± 7.97 kg. The patients were comparable in terms of height and weight. This is in accordance with the studies conducted by Mostafa et al., Ismail et al., Ayutuluk et al., and Mooen et al. [18,21,23,24].

Adequate pain control and postoperative pain assessment become complicated with a higher incidence of coexisting diseases and concurrent medications. Among the study population, 28 patients (93.33%) in group B and 25 patients (83.33%) in group D belonged to ASA class I, and two patients (6.67%) in group B, five patients (16.67%) in group D belonged to ASA class II. The p-value is 0.424 and holds no statistical significance. This is similar to the study conducted by Salem et al. (p 0.34). The studies done by Mostafa et al., Ismail et al., Ayutuluk et al., Mooen et al., and Devi et al. [18,21,23-25] show a similar finding that ASA class is not statistically significant between the groups.

In our study, the mean duration of surgery in group B was 109.43 ± 9.97 min, and in group D was 111.03 ± 10.06 min. The same surgeon did all surgeries. Sixteen patients from both groups had a surgery time between 100 and 120 min. Patients in both groups were comparable based on the mean duration of surgery. The p-value of 0.538 holds no statistical significance. The study by Mostafa et al. concluded that the time of surgery in the studied groups was not statistically significant, similar to ours [18].

The two-segment regression time was measured in our study to estimate the time by which the effect of spinal anaesthesia would wear off. This was done to justify the prolongation of the analgesia due to intra-articular drug administration and not spinal anaesthesia. Most patients in both groups had two-segment regression between 81 and 85 min (15 in group B, 14 in group D). The mean regression time in group B was 80.56 ± 3.82 min, and in group D was 81.13 ± 3.25 min. The p-value was 0.538, signifying that the regression in both groups was comparable. This is in accordance with the study by Moeen et al. [21].

Limitations

We did not compare Dexmedetomidine with its other routes of administration like epidural, intrathecal or intravenous, nor check for plasma levels of locally administered study drugs for toxicity. We did not take long-term follow-ups of the patients whether any local tissue damage (Chondrolysis) occurred in the intra-articular space due to the administration of study drugs.

## Conclusions

From our study, we would like to conclude that Dexmedetomidine added to Intra-articularly administered Bupivacaine prolongs the duration of postoperative analgesia, thereby providing better analgesic quality and decreasing the total dose of analgesics required without significant side effects. Thus, we suggest this combination is safe and effective for managing postoperative pain following arthroscopic surgeries of the knee.
